# Quantum Reactivity: An Indicator of Quantum Correlation

**DOI:** 10.3390/e22010006

**Published:** 2019-12-19

**Authors:** Shahabeddin M. Aslmarand, Warner A. Miller, Verinder S. Rana, Paul M. Alsing

**Affiliations:** 1 Department of Physics, Florida Atlantic University, Boca Raton, FL 33431, USA; wam@fau.edu; 2 Naval Information Warfare Center Pacific (NIWC PAC), San Diego, CA 92152, USA; verinder.rana@navy.mil; 3 Information Directorate, Air Force Research Laboratory, Rome, NY 13441, USA; paul.alsing@us.af.mil

**Keywords:** entanglement monotones, quantum information, quantum entanglement, quantum correlations, quantum information geometry

## Abstract

Geometry is often a valuable guide to complex problems in physics. In this paper, we introduce a novel geometric quantity called quantum reactivity (QR) to probe quantum correlations in higher-dimensional quantum systems. Much like quantum discord, QR is not a measure of quantum entanglement but can be useful in quantum information processes where a notion of quantum correlation in higher dimensions is needed. Both quantum discord and QR are extendable to an arbitrarily large number of qubits; however, unlike discord, QR satisfies the invariance under unitary operations. Our approach parallels Schumacher’s singlet state triangle inequality, which used an information geometry-based entropic distance. We use a generalization of information distance to area, volume, and higher-dimensional volumes and then use these to define a quantity that we call QR, which is the familiar ratio of surface area to volume. We examine a spectrum of multipartite states (Werner, W, GHZ, randomly generated density matrices, etc.) and demonstrate that QR can provide an ordering of these quantum states as to their degree of quantum correlation.

## 1. Toward Understanding Multipartite Quantum Correlations

Entanglement in quantum systems is widely studied as a resource for quantum computing [1]. However, quantum correlation and the notion of distinguishability is arguably crucial, e.g., quantum non-locality without entanglement and non-entangled states can be used in quantum computing applications [2,3,4,5,6,7]. In particular, it is demonstrated that separable states can potentially scale more favorably than classically allowed [8]. Therefore, in this manuscript, we will concentrate on the broader class of quantum correlation rather than quantum entanglement. The utility of quantum correlation is revealed in quantum cryptography, quantum dense coding, and quantum teleportation [9,10]. Furthermore, the quantum correlation has the potential to optimize classical communication by reducing its complexity [11]. For example, the computational power of a quantum network is related to its degree of quantum correlation [2]. Unfortunately, the quantum correlation has an exponentially increasing complex structure—correlations exist in a Hilbert space of exponentially large dimension [12] and, although many measures of quantum correlation have been proposed, we are far away from having a satisfying measure of quantum correlation for any system with more than two qubits. The goal of this paper is not to offer a new measure of correlation but rather to probe the relationship between quantum correlation in quantum systems and its information geometry. We offer a new quantity that refer to as quantum reactivity (QR) that is similar to quantum discord (QD) [13]. While QR and QD are not a measures of quantum entanglement, nevertheless they can be useful for some quantum information processes.

We will briefly introduce Zurek and Oliver’s definition of QD since our definition of QR and QD share a lot of similarities. QD is defined for two-qubit systems and arises from the difference in expressions of mutual information in quantum mechanical systems

(1) $$I\left(\rho_{AB}\right):=S\left(\rho_{A}\right)+S\left(\rho_{B}\right)-S\left(\rho_{AB}\right)$$

(2) $$J_{A}\left(\rho_{AB}\right):=S\left(\rho_{B}\right)-S\left(\rho_{AB|\Pi^{A}}\right),$$

where *S* is the von Neumann entropy, $\rho_{AB}$ is the joint density matrix of the bipartite system for *A* and *B* and $\rho_{A}=Tr_{B}\left(\rho_{AB}\right)$ is the reduced density matrix. Additionally, $S(\rho_{AB|\Pi^{A}})$ is the von Neumann entropy of the system when the results of the measurements on subsystem *A* are $\Pi^{A}$. The mutual informations, *J* and *I*, are not ordinarily equal. This disparity led to the definition of QD

(3) $$D_{A}\left(\rho \right):=I\left(\rho \right)-\max_{\left\{\Pi_{j}^{A}\right\}}J_{\left\{\Pi_{j}^{A}\right\}}\left(\rho \right).$$

Rulli and Sarandy [14] further developed this measure around multipartite systems. They achieved this by asymmetric extension of the bipartite QD and called it global quantum discord (GQD). GQD is non-negative and symmetric under subsystem exchange. However, one can easily show that QD is not invariant under local unitary transformation, nor it is monotonic under local operations and classical communication (LOCC) [15], and GQD inherits these issues from QD [16]. Given these issues, and that it can be created by local means, one can argue that this quantity is not useful for quantum information processing. Nevertheless, QD has some operational meaning for some quantum applications.

From what we have seen in the previous paragraph, one can argue that for operational purposes, and depending on the type of quantum application, we can have a notion of quantum correlation without having a measure of quantum correlation. This simplifies the problem by allowing one to loosen the conditions that this quantity has to satisfy. Using this, and combining it with the fact that often in a complex physical system the geometry can give us a good handle on an understanding of the problem, we offer a novel geometrical quantity as a notion of quantum correlation that is invariant under unitary transformation. Our approach parallels Schumacher’s [17] singlet state triangle inequality, which used an information geometry-based entropic distance introduced by Rokhlin [18] and Rajski [19] to derive an inequality that captures the non-classical nature of quantum systems and can be experimentally realized in a quantum optics laboratory from the clicks on the detector setting. Schumacher’s geometrical approach to quantum correlation enables him to capture entanglement of the singlet state through a Shannon-based entropic measure of distance. Schumacher managed to capture entanglement using Shannon’s entropy by introducing extra detectors for each of the two entangled qubits. Alice measures one of the two qubits by choosing randomly between the two detectors ($A_{1}$ and $A_{2}$), Bob does the same ($B_{1}$ and $B_{2}$). This enabled Schumacher to construct a quadrilateral and demonstrate, that for certain settings of the detectors, the direct distance between $A_{1}$ and $B_{2}$ is longer than the sum of the three other indirect distances. Schumacher demonstrated that quantum correlation is related to geometry.

Following Schumacher’s footsteps, we defined QR for a bipartite system as

(4) $$R:=\frac{1}{{\langle D_{AB}\rangle }_{{}_{M}}}.$$

where, $D_{AB}$ is the information distance between the two qubits in a measurement setting, defined in detail in Section 2 in Equation (11), and the average, ${\langle \rangle }_{M}$, over the space of all detector settings of *A* and *B* is defined in Section 4 in Equation (51).

We generalize this to multipartite systems by a recent extension of the information distance to information area and higher-dimensional volumes [20,21]. This allows us to define QR as the average of the bounding surface area divided by volume, each averaged over all possible measurement basis, ${\langle \rangle }_{M}$. In this way QR can be sensitive to quantum correlations while still using Shannon-based entropic distances defined over the four measurement outcomes. We find that there is relationship between QR and quantum correlation and QR is invariant under unitary LOCC [22]. The remainder of this paper will introduce QR. In Section 2, we describe a generalization of Schumacher’s geometry to higher-dimensional multipartite quantum states. In Section 3, we provide an illustrative example of a generalization of the Schumacher geometry for the Greenberger– Horne–Zeilinger |GHZ〉 tripartite state [23]. In Section 4, we define QR for a multipartite state, and we apply this to a spectrum of multipartite states (bipartite and tripartite Werner state, four-qubit Werner state, as well as a modified Werner state). Finally, in Section 6 we discuss future questions that we will explore by using QR.

## 2. Quantum Geometry in the Space of Measurements

**Figure 1:**
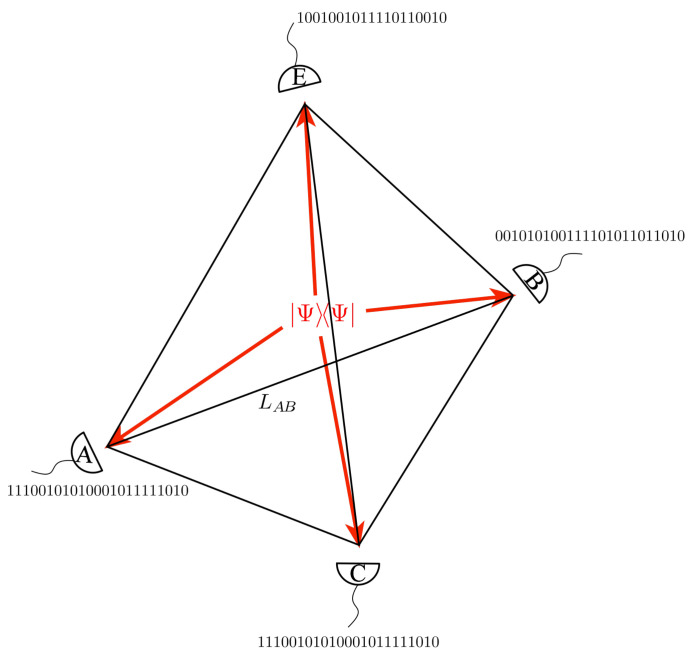
An illustration of a simplicial geometry representation of a four-qubit state illustrated by the four photons emanating from the center of the tetrahedron (arrows). The tetrahedral geometry emerges from a recording of measurements made by *A*, *B*, *C*, and *E* over an ensemble of identically prepared states. At each observer, their string of 1’s and 0’s form a binary random variable and the combination forms a joint probability distribution from which we can define the complete geometry of the tetrahedron. Here the tetrahedron is parameterized by the eight Stokes parameters of the detectors of our four observers.

The assertion of John Archibald Wheeler that “no elementary quantum phenomenon is a phenomenon until it is brought to close by an irreversible act of amplification” was inspired by Niels Bohr. This point of view, together with the principle of complementarity, is at the very heart of Wheeler’s ’It-from-Bit’ framework [24]. Here we explore a spectrum of quantum networks within this information-centric geometric landscape. The quantum network is a multipartite state with *d* qubits that can contain entanglement and be mixed. Observers create a “space of measured data” from repeated experiments over a set of identically prepared quantum states. Each observer records a “1” if their detector triggers, otherwise a “0" is recorded. This generates a string of 1’s and 0’s at each detector, as illustrated in Figure 1. The string of numbers can be represented by a binary random variable. The observers may have more than one detector, and therefore each observer may acquire more than one binary random variable. Once these random variables are formed, we can apply an information geometry measure of distance, area, volume, and *d*-volumes to the network of observers [18,19,21,25]. Following Schumacher, these measures are defined using the familiar Shannon expression for conditional entropy that will be described in this section [17,26].

What is unique to our definition of quantum information geometry is that we project the qudit state into a classical space of a *d*-dimensional joint probability distribution. We make repeated measurements on an ensemble of identically prepared quantum states to produce this probability distribution. From this distribution, we can calculate all other marginal or joint probability distributions. In this sense we are constructing a functional from the quantum density matrix to a classical distribution; we are essentially “probing the quantum state within a space of measurements.” [20] We describe this in detail below after we discuss novel entropic geometry constructions.

We construct our reactivity based on information geometry [21]. The fundamental quantity of any information theory is information entropy. The entropy, H(X) is a function of the probability spectrum of values of a random variable *X*. Similar to Schumacher, we use Shanon’s entropy [26] where

(5) $$H\left(X\right)=-\sum_{i=1}^{s}p\left(x_{i}\right)\ln p(x_{i}),$$

for an *s*-state random variable. Here $p\left(x_{i}\right)=p\left(X\;=\;x_{i}\right)$ is the probability that the random variable *X* has the value $x_{i}$. Probability measures uncertainty about the occurrence of a single event, but entropy provides a measure of the uncertainty of a collection of events. In other words, the H(X) is a measure of the uncertainty associated with the probability distribution over *X*.

The entropy is the largest when our uncertainty of the value of the random variable is complete (e.g., uniform distribution of probabilities), and the entropy is zero if the random variable always takes on the same value. These bound the possible values for entropy,

(6) $$0\leq H\left(X_{i}\right)\leq log\left(s\right).$$

For example, consider an electron with two possible configurations, up and down. The most general quantum state is the normalized superposition state

(7) $$|\psi \rangle =c_{1}|\;↑\rangle +c_{2}|\;↓\rangle$$

(8) $$P\left(x_{i}\right)=\left\{\begin{array}{ccc} ↑ & \mathrm{with}\;\mathrm{probability} & {|c_{1}|}^{2}\\ ↓ & \mathrm{with}\;\mathrm{probability} & {|c_{2}|}^{2} \end{array}\right..$$

The entropy of this two-state probability distribution is

(9) $$H\left(X\right)=-\sum_{i=1}^{2}P\left(x_{i}\right)\log_{2}P\left(x_{i}\right)=-{|c_{1}|}^{2}\log_{2}\left({|c_{1}|}^{2}\right)-{|c_{2}|}^{2}\log_{2}\left({|c_{2}|}^{2}\right).$$

In the case that ${|c_{2}|}^{2}={|c_{1}|}^{2}=\frac{1}{2}$, H(X) will equal to 1. This entropy obeys a set of special properties for an *s*-state random variable:

1. H(X)≥0, with equality if one outcome has probability 1.
2. H(X)≤lns, where *s* is the number of possible outcomes for X, with equality if each outcome has probabilities 1/s.
3. H(X,Y)≥H(X), where H(X,Y) is the joint information for *X* and *Y*.
4. H(X,Y)≤H(X)+H(Y) with equality if *X* and *Y* are independent.

These properties make it possible to define an information geometry measure. It is also useful to introduce the conditional entropy

(10) H(X|Y)=H(X,Y)−H(Y)

that measures the uncertainty of *X* after *Y* is known. We use these entropies to build our geometrical structure. There are many possible definitions; however, in this paper, we follow Schumacher, and use an extension of the Shannon-based information distance defined by Rokhlin [18] and Rajski [19] (RR) where

(11) $$D_{AB}=H_{A|B}+H_{B|A}=2H_{AB}-H_{A}-H_{B}.$$

This information distance is a proper metric since,

1. It is constructed to be symmetric, $D_{AB}=D_{BA}$
2. It obeys the triangle inequality, $D_{AB}\geq D_{AC}+D_{CB}$.
3. It is non-negative, $D_{AB}\geq 0$, and equal to 0 when *A*“=”*B*.

In the case that *A* and *B* are uncorrelated

(12) $$H_{AB}=H_{A}+H_{B}$$

and the distance obtains its maximal permissible value

(13) $$D_{AB}=H_{A}+H_{B}.$$

Our choice of metric is not unique. Different choices can give distinct behaviors [27]. In this manuscript, we use the RR metric.

In addition to the information distance, we can analogously assign an information area to the geometry of measurement space, [20,21] where

(14) $$A_{ABC}=H_{A|BC}H_{B|CA}+H_{B|CA}H_{C|AB}+H_{C|AB}H_{A|BC}.$$

This can be generalized to higher-dimensional simplexes, e.g., the information volume for a tetrahedron

(15) $$\begin{array}{cc} V_{ABCD} & :=H_{A|BCD}H_{B|CDA}H_{C|DAB}+H_{B|CDA}H_{C|DAB}H_{D|ABC}\\ \; & \;\;+H_{C|DAB}H_{D|ABC}H_{A|CDB}+H_{D|ABC}H_{A|BCD}H_{B|CDA} \end{array}$$

is a natural generalization of Equation (14) [20,21]

For classical probability distributions, we showed that these formulas are well defined and have all of the requisite symmetries, positivity, bounds, and structure usually required for such formula [21]. Each of these geometric measures are bounded,

(16) $$0\leq D_{AB}\leq H_{A}+H_{B}\leq 2\log s,$$

(17) $$0\leq A_{ABC}\leq 3{\left(\log s\right)}^{2},$$

(18) $$0\leq V_{ABCD}\leq 4{\left(\log s\right)}^{3}.$$

The minimum values occur when the random variables are completely correlated, and their maximum values obtained when the random variables are completely uncorrelated.

We use the information distance, area and volume defined on the “space of measurements” to define a curvature-based definition of quantum correlation that we refer to as reactivity. We have found that such curvature measures provide a relationship between the curvature of the “space of measurements” and the quantum correlation of the underlying quantum system. We find that quantum entanglement is reactive.

## 3. An Illustrative Example: The Information Geometry of a Three-Qubit State

We find it useful to introduce our approach by focusing on the maximally entangled qutrit state, namely

(19) $$|GHZ\rangle =\frac{1}{\sqrt{2}}\left(|111\rangle +|000\rangle \right).$$

For this state, we can calculate the information geometry using Equations (11)–(15). This is a three-qubit state. One qubit is sent to Alice (*A*), one to Bob (*B*), and the third to Charlie (*C*). *A*, *B* and *C* prepare a large ensemble (*N* copies) of identically prepared states Equation (19), The three observers each choose a measurement basis. They then measure the initial state (Equation (19) with their choice of detector settings. The observers record their digital measurements. In particular, they record a “1" if their detector registers an event; otherwise they record a “0" and keep their order in time. This procedure produces an ordered string of 0’s and 1’s of length *N*. After these measurements, *A*, *B*, and *C*, each have generated a binary random variable. Together, *A*, *B*, and *C* form a joint probability distribution,

(20) $$p\left(A=a_{i},B=b_{i},C=c_{i}\right)\;\;\mathrm{with}\;a_{i},b_{i},c_{i}\in \left\{0,1\right\}.$$

This gives the probability that *A*’s detector, *B*’s detector and *C*’s detector will measure the values $a_{i}$, $b_{i}$ and $c_{i}$, respectively. From this joint distribution we can define entropy and extract the information geometry. We can calculate the three information distances as well as the information area formed by *A*, *B* and *C*.

Let us suppose *A* oriented her detector at the Stokes angles $\theta_{a}$ and $\varphi_{a}$, that *B* oriented his apparatus at angles $\theta_{b}$ and $\varphi_{b}$ and *C* oriented his apparatus at angels $\theta_{c}$ and $\varphi_{c}$. The measurement operators for each of these detectors are

(21) $$M_{A}=\frac{1}{2}\left(I+\cos\left(\theta_{a}\right)\sigma_{z}+\sin\left(\theta_{a}\right)\sin\left(\varphi_{a}\right)\sigma_{y}+\sin\left(\theta_{a}\right)\cos\left(\varphi_{a}\right)\sigma_{x}\right)⊗I⊗I,$$

(22) $$M_{B}=I⊗\frac{1}{2}\left(I+\cos\left(\theta_{b}\right)\sigma_{z}+\sin\left(\theta_{b}\right)\sin\left(\varphi_{b}\right)\sigma_{y}+\sin\left(\theta_{b}\right)\cos\left(\varphi_{b}\right)\sigma_{x}\right)⊗I,$$

(23) $$M_{C}=I⊗I⊗\frac{1}{2}\left(I+\cos\left(\theta_{c}\right)\sigma_{z}+\sin\left(\theta_{c}\right)\sin\left(\varphi_{c}\right)\sigma_{y}+\cos\left(\varphi_{c}\right)\sin\left(\theta_{c}\right)\sigma_{x}\right).$$

These measurements directly yield the joint probability (Equation (20), as well as all the pairwise probabilities, e.g., $p(A=a_{i},B=b_{i})$ and individual probabilities, e.g., $p(A=a_{i})$. This, in turn, is sufficient to determine all the entropies and conditional entropies. Without loss of generalization, we choose to simplify our expressions by fixing *A*’s detector to the Stokes parameters $\theta_{a}=\varphi_{a}=0$. The eight joint probabilities from the three measurements on this entangled state $M_{A}⊗M_{B}⊗M_{C}|GHZ\rangle$ are

(24) $$\begin{array}{ccc} p(A=1,B=1,C=1) & = & \frac{1}{2}\cos^{2}\left(\theta_{b}\right)\cos^{2}\left(\theta_{c}\right), \end{array}$$

(25) $$\begin{array}{ccc} p(A=0,B=1,C=1) & = & \frac{1}{2}\sin^{2}\left(\theta_{b}\right)\sin^{2}\left(\theta_{c}\right), \end{array}$$

(26) $$\begin{array}{ccc} p(A=1,B=1,C=0) & = & \frac{1}{2}\cos^{2}\left(\theta_{b}\right)\sin^{2}\left(\theta_{c}\right), \end{array}$$

(27) $$\begin{array}{ccc} p(A=0,B=1,C=0) & = & \frac{1}{2}\sin^{2}\left(\theta_{b}\right)\cos^{2}\left(\theta_{c}\right), \end{array}$$

(28) $$\begin{array}{ccc} p(A=1,B=0,C=1) & = & \frac{1}{2}\sin^{2}\left(\theta_{b}\right)\cos^{2}\left(\theta_{c}\right), \end{array}$$

(29) $$\begin{array}{ccc} p(A=0,B=0,C=1) & = & \frac{1}{2}\cos^{2}\left(\theta_{b}\right)\sin^{2}\left(\theta_{c}\right), \end{array}$$

(30) $$\begin{array}{ccc} p(A=1,B=0,C=0) & = & \frac{1}{2}\sin^{2}\left(\theta_{b}\right)\sin^{2}\left(\theta_{c}\right), \end{array}$$

(31) $$\begin{array}{ccc} p(A=0,B=0,C=0) & = & \frac{1}{2}\cos^{2}\left(\theta_{b}\right)\cos^{2}\left(\theta_{c}\right) \end{array}$$

Tracing these joint probabilities over each observer yields the twelve pairwise joint probabilities

(32) $$\begin{array}{ccc} p(A=1,B=1) & = & \frac{1}{2}\cos^{2}\left(\theta_{b}\right), \end{array}$$

(33) $$\begin{array}{ccc} p(A=1,B=0) & = & \frac{1}{2}\sin^{2}\left(\theta_{b}\right), \end{array}$$

(34) $$\begin{array}{ccc} p(A=1,C=1) & = & \frac{1}{2}\cos^{2}\left(\theta_{c}\right), \end{array}$$

(35) $$\begin{array}{ccc} p(A=1,C=0) & = & \frac{1}{2}\sin^{2}\left(\theta_{c}\right), \end{array}$$

(36) $$\begin{array}{ccc} p(A=0,B=1) & = & \frac{1}{2}\sin^{2}\left(\theta_{b}\right), \end{array}$$

(37) $$\begin{array}{ccc} p(A=0,B=0) & = & \frac{1}{2}\cos^{2}\left(\theta_{b}\right), \end{array}$$

(38) $$\begin{array}{ccc} p(A=0,C=1) & = & \frac{1}{2}\sin^{2}\left(\theta_{c}\right), \end{array}$$

(39) $$\begin{array}{ccc} p(A=0,C=0) & = & \frac{1}{2}\cos^{2}\left(\theta_{c}\right), \end{array}$$

(40) $$\begin{array}{ccc} p(B=1,C=1) & = & \frac{1}{2}\left(\cos^{2}\left(\theta_{b}\right)\cos^{2}\left(\theta_{c}\right)+\sin^{2}\left(\theta_{b}\right)\sin^{2}\left(\theta_{c}\right)\right), \end{array}$$

(41) $$\begin{array}{ccc} p(B=1,C=0) & = & \frac{1}{2}\left(\cos^{2}\left(\theta_{b}\right)\sin^{2}\left(\theta_{c}\right)+\sin^{2}\left(\theta_{b}\right)\cos^{2}\left(\theta_{c}\right)\right), \end{array}$$

(42) $$\begin{array}{ccc} p(B=0,C=1) & = & \frac{1}{2}\left(\sin^{2}\left(\theta_{b}\right)\cos^{2}\left(\theta_{c}\right)+\cos^{2}\left(\theta_{b}\right)\sin^{2}\left(\theta_{c}\right)\right), \end{array}$$

(43) $$\begin{array}{ccc} p(B=0,C=0) & = & \frac{1}{2}\left(\cos^{2}\left(\theta_{b}\right)\cos^{2}\left(\theta_{c}\right)+\sin^{2}\left(\theta_{b}\right)\sin^{2}\left(\theta_{c}\right)\right). \end{array}$$

Finally, tracing the joint probability over all pairs of observers gives us the six probabilities for the measurement outcomes of *A*, *B* and *C* as

(44) $$p\left(A=a_{i}\right)=p\left(B=b_{i}\right)=p\left(C=c_{i}\right)=\frac{1}{2}.$$

From these probabilities we find the entropies. The joint entropy $H_{ABC}$ of our three observers is

(45) $$\begin{array}{ccc} H_{ABC}=1 & - & \cos^{2}\left(\theta_{b}\right)\cos^{2}\left(\theta_{c}\right)\log\left(\cos^{2}\left(\theta_{b}\right)\cos^{2}\left(\theta_{c}\right)\right)\\ & - & \cos^{2}\left(\theta_{b}\right)\sin^{2}\left(\theta_{c}\right)\log\left(\cos^{2}\left(\theta_{b}\right)\sin^{2}\left(\theta_{c}\right)\right)\\ & - & \sin^{2}\left(\theta_{b}\right)\cos^{2}\left(\theta_{c}\right)\log\left(\sin^{2}\left(\theta_{b}\right)\cos^{2}\left(\theta_{c}\right)\right)\\ & - & \sin^{2}\left(\theta_{b}\right)\sin^{2}\left(\theta_{c}\right)\log\left(\sin^{2}\left(\theta_{b}\right)\sin^{2}\left(\theta_{c}\right)\right). \end{array}$$

The three joint entropies between each pair of our observers are

(46) $$\begin{array}{ccc} H_{AB} & = & 1-\sin^{2}\left(\theta_{b}\right)\log\left(\sin^{2}\left(\theta_{b}\right)\right)-\cos^{2}\left(\theta_{b}\right)\log\left(\cos^{2}\left(\theta_{b}\right)\right), \end{array}$$

(47) $$\begin{array}{ccc} H_{AC} & = & 1-\sin^{2}\left(\theta_{c}\right)\log\left(\sin^{2}\left(\theta_{c}\right)\right)-\cos^{2}\left(\theta_{c}\right)\log\left(\cos^{2}\left(\theta_{c}\right)\right), \end{array}$$

(48) $$\begin{array}{ccc} H_{BC} & = & 1-\left(\cos^{2}\left(\theta_{b}\right)\cos^{2}\left(\theta_{c}\right)+\sin^{2}\left(\theta_{b}\right)\sin^{2}\left(\theta_{c}\right)\right)\\ & & \log\left(\cos^{2}\left(\theta_{b}\right)\cos^{2}\left(\theta_{c}\right)+\sin^{2}\left(\theta_{b}\right)\sin^{2}\left(\theta_{c}\right)\right)\\ & & \;\;\;-\left(\sin^{2}\left(\theta_{b}\right)\cos^{2}\left(\theta_{c}\right)+\cos^{2}\left(\theta_{b}\right)\sin^{2}\left(\theta_{c}\right)\right)\\ & & \log(\left(\sin^{2}\left(\theta_{b}\right)\cos^{2}\left(\theta_{c}\right)+\cos^{2}\left(\theta_{b}\right)\sin^{2}\left(\theta_{c}\right)\right), \end{array}$$

and finally the three entropies of our observers are equal

(49) $$H_{A}=H_{B}=H_{C}=1.$$

The lengths of the three edges of the triangle formed by *A*, *B* and *C* for this |GHZ〉 state can be calculated using these entropies and Equation (11). Similarly, the entropic area is defined using Equation (14) and the relation for conditional entropy in Equation (10).

We use the same procedure when we calculate the information geometry for the two-qubit and three-qubit Werner state in the next section, as well as for the four-qubit Werner state. The procedure is the same for higher-dimensional multipartite states.

## 4. Quantum Reactivity as a Notion for Quantum Correlation

In Section 1 we made reference to QD as measures of quantum correlation for two-qubit states, and we highlighted a problem with it. In this section, we define our geometric notion of correlation that we call QR. We also show that QR yields result that is qualitatively similar to the GQD. We demonstrate this for a spectrum of known states. However, unlike QD and GQD, our measure is invariant under unitary operation.

Reactivity of a chemical agent is naturally defined as the ratio of its surface area to volume and is related to its curvature. We adopt the same definition and apply it to the information area and volumes outlined in Section 2 through Equations (14) and (15) and their extensions to higher dimensions in [21]. In particular, a natural definition of curvature-based reactivity can be defined for a *d*-qubit simplistic geometry as

(50) $$R:=\frac{{\langle {}^{{}^{\left(d-2\right)}}\;A\rangle }_{{}_{M}}}{{\langle {}^{{}^{\left(d-1\right)}}\;V\rangle }_{{}_{M}}}.$$

The brackets, ${\langle {}^{{}^{\left(d\right)}}\;V\rangle }_{{}_{M}}$, denote the volume-averaged mean of the information volume, ${}^{{}^{\left(d\right)}}\;V$, over all possible measurements M. In particular

(51) $${\langle {}^{{}^{\left(d\right)}}\;V\rangle }_{{}_{M}}:=\frac{1}{V_{\;{}_{M}}}\left(\int_{M}{}^{{}^{\left(d\right)}}\;V\right),$$

and $V_{M}={\langle 1\rangle }_{{}_{M}}={\left(2\pi^{2}\right)}^{d}$ is the volume of the “space of measurements.” Here we have, without loss of generalization, fixed one of the detectors at a fixed point on its Bloch sphere. We integrate over all measurement settings on the remaining (d−1) qubits.

Our proposed notion of quantum correlation is invariant under unitary operation.

**Theorem** **1.** *The QR of an n qubit system is invariant under local unitary operator.*

**Proof.**  In the case of a two qubit system QR is defined in terms of the reciprocal of the information distance,

(52) $${\langle D_{AB}\rangle }_{{}_{M}}=\frac{1}{V_{\;{}_{M}}}\left(\int_{M}D_{AB}\right):=\frac{1}{2\pi^{2}}\int_{0}^{\pi }\int_{0}^{2\pi }D_{AB}\left(\theta_{b},\varphi_{b}\right)\;d\theta_{b}d\varphi_{b}.$$

It is well know that one can write any unitary operator as

(53) $$U=e^{i\alpha }\left(\begin{array}{cc} e^{-i\frac{\beta }{2}} & 0\\ 0 & e^{i\frac{\beta }{2}} \end{array}\right)\left(\begin{array}{cc} cos\frac{\gamma }{2} & -sin\frac{\gamma }{2}\\ sin\frac{\gamma }{2} & cos\frac{\gamma }{2} \end{array}\right)\left(\begin{array}{cc} e^{-i\frac{\delta }{2}} & 0\\ 0 & e^{i\frac{\delta }{2}} \end{array}\right)$$

which are three consecutive rotations. Then the conditional entropy can be written as

(54) $$H_{A|B}=-\sum Tr\left(M_{A}^{t}⊗M_{B}^{t}U\rho U^{t}M_{A}⊗M_{B}\right)\log\frac{Tr(M_{A}^{t}⊗M_{B}^{t}U\rho U^{t}M_{A}⊗M_{B})}{Tr(I⊗M_{B}^{t}U\rho U^{t}I⊗M_{B})},$$

which is equal to

(55) $$H_{A|B}=-\sum Tr\left(U^{t}M_{A}⊗M_{B}M_{A}^{t}⊗M_{B}^{t}U\rho \right)\log\frac{Tr(U^{t}M_{A}⊗M_{B}M_{A}^{t}⊗M_{B}^{t}U\rho )}{Tr(U^{t}I_{A}⊗M_{B}I_{A}⊗M_{B}^{t}U\rho )}.$$

In other words, applying the unitary transformation to the initial density matrix is equivalent to rotating our measurement operators by a constant angle from $\theta \Rightarrow \theta^{{}^{\prime }}$ and $\phi \Rightarrow \phi^{{}^{\prime }}$, with

(56) $$\begin{array}{cc} \; & \theta =\theta^{{}^{\prime }}+c,\\ \; & \phi =\phi^{{}^{\prime }}+c^{\prime }, \end{array}$$

and where *c* and $c^{\prime }$ are constants. Therefore,

(57) $$D_{AB}\left(\theta_{b},\varphi_{b}\right)\Rightarrow D_{AB}\left(\theta_{b}^{{}^{\prime }},\varphi_{b}^{{}^{\prime }}\right),$$

and

(58) $${\langle D_{AB}\rangle }_{{}_{M}}=\frac{1}{V_{\;{}_{M}}}\left(\int_{M}D_{AB}\right):=\frac{1}{2\pi^{2}}\int_{0}^{\pi }\int_{0}^{2\pi }D_{AB}\left(\theta_{b}^{{}^{\prime }},\varphi_{b}^{{}^{\prime }}\right)\;d\theta_{b}d\varphi_{b}.$$

Now one can make the variable change from the original frame to prime frame and get to the original form of QR. The same is true in the case of *n* qubit system since we are averaging over all possible sets of measurements, thus rotating our frame will not change the value of QR. □

In reference [22] we have proved that, in the case of 2 qubit systems, our measure is also non-increasing under local operations and classical communication. However, currently we do not have the proof that the same is true for any *n* qubit system.

In the next three subsections, we examine the QR for the following states: (1) The mixed bipartite Werner state; (2) the mixed three-qubit Werner state and a less-correlated modified Werner state; and finally (3) the four-qubit generalization of the Werner state.

### 4.1. Reactivity of a Bipartite Werner State

For illustrative purposes, we first consider the QR of the bipartite Werner state. This state has a controllable parameter, λ, that governs the degree of entanglement and quantum correlation

(59) $$\rho_{{}_{Werner}}=\lambda \;|\psi_{singlet}\rangle \langle \psi_{singlet}|+\frac{1-\lambda }{4}\;I.$$

This state is separable when λ≤1/3, totally mixed when λ=0 and the maximally entangled singlet state when λ=1 [28].

We imagine that an observer Alice (*A*) makes measurements on one of the qubits, and Bob (*B*) makes measurements on the other qubit. Without loss of generalization we can permanently fix the Stokes parameters of *A* to zero, i.e., $\theta_{a}=\varphi_{a}=0$. However, *B* makes measurements over his Bloch sphere with each measurement characterized by its two Stokes angles $\theta_{b}$ and $\varphi_{b}$. The QR is defined on the emergent geometry of the space of measurements. While QR may be more intuitive when applied to a four-qubit state, nevertheless we can apply this to a two-qubit state equally well by using Equation (50) with d=2, where we find

(60) $$R:=\frac{1}{{\langle D_{AB}\rangle }_{{}_{M}}}.$$

Here the weighted average of the distance is defined over the two-dimensional Bloch–sphere measurements,

(61) $${\langle D_{AB}\rangle }_{{}_{M}}=\frac{1}{V_{\;{}_{M}}}\left(\int_{M}D_{AB}\right):=\frac{1}{2\pi^{2}}\int_{0}^{\pi }\int_{0}^{2\pi }D_{AB}\left(\theta_{b},\varphi_{b}\right)\;d\theta_{b}d\varphi_{b}.$$

and yields the QR defined over all possible relative combinations of detector orientations (M) for *A* and *B*.

**Figure 2:**
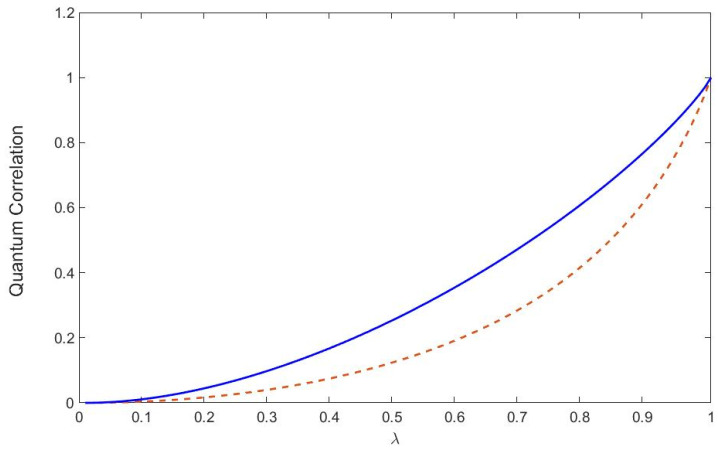
Quantum reactivity (QR; **dashed line**) and global quantum discord (GQD; **solid line**) are plotted as a function of the entanglement parameter λ for the two-qubit Werner state defined by Equation (59).

Each value λ of the entanglement parameter in Equation (59) yields a unique value for the quantum reactivity, R. The function R(λ) with respect to correlation parameter λ is a monotonically increasing function as illustrated in Figure 2. Its minimum is the totally mixed state $\rho_{\;{}_{Werner}}\left(\lambda =0\right)=\frac{1}{4}I$, and it obtains its max value at λ=1 when the state is the maximally entangled singlet state, $|\psi_{singlet}\rangle$. As our state gets more correlated as λ increases the geometry of the space of measurements is more reactive (or curved).

The behavior of the QR shares the essential characteristics of Zurek and Ollivier’s QD measure as illustrated in Figure 2. We plotted a linear transformation of the QR to make R(0)=0 (totally mixed)and R(1)=1 (maximally entangled bipartite state). Both QD and QR yield non-zero quantum correlations even below λ=1/3 when concurrence shows us that there is no entanglement [29]. Quantum correlation can still exist even inseparable states that are not entangled can contain quantum correlations, and this stronger than classical correlation may be advantageous for some quantum computing applications, e.g., quantum non-locality without entanglement [2,3,6,7]. In addition, it was demonstrated that separable states can potentially scale more favorably than classically allowed [8]. We emphasize that even though the behavior of QD and reactivity are qualitatively similar, the two measures of quantum correlation are fundamentally different, and this difference may be useful.

### 4.2. Reactivity of the Tripartite Werner State and Modified Werner State

In this section, we explore the behavior of reactivity for two different tripartite states, the Werner state, and a modified Werner state. Both states are mixed states. The density matrix for the three-qubit Werner state

(62) $$\rho_{1}=\lambda \;|GHZ\rangle \langle GHZ|+\frac{1-\lambda }{8}\;I$$

is constructed as a weighted sum of the density matrix for the maximally entangled |GHZ〉 state

(63) $$|GHZ\rangle =\frac{1}{\sqrt{2}}\left(|000\rangle +|111\rangle \right),$$

and the totally mixed state *I*. When λ=0 the state is the separable mixed state with least correlation and when λ=1 it is the maximally entangled state. The parameter λ is the entanglement parameter. We compare the QR for this Werner state and a modified Werner state. The density matrix for the modified Werner state is based on the less-entangled |W〉 state [30], where

(64) $$\rho_{2}=\lambda \;|W\rangle \langle W|+\frac{1-\lambda }{8}\;I.$$

Here too, this density matrix is constructed as a weighted sum of the density matrix for the less-entangled |W〉 state

(65) $$|W\rangle =\frac{1}{\sqrt{3}}\left(|001\rangle +|010\rangle +|100\rangle \right),$$

and the uncorrelated mixed state *I*. When λ=0 the state is the maximally mixed state and when λ=1 it is the highly entangled |W〉 state.

The three-qubit reactivity is equally well defined over the “space of measurements” on an ensemble of identically prepared quantum states made by three observers, *A*, *B* and *C*. The QR is defined by Equation (50) by setting d=3. The three observers form a triangle. As they record their measurements from an ensemble of identically prepared quantum states they will generate a joint probability distribution

(66) $$p\left(A=a_{i},B=b_{i},C=c_{i}\right),$$

from whence we can calculate all the entropies ($H_{A}$), joint entropies ($H_{AB}$ and $H_{ABC}$) and conditional entropies ($H_{A|B}$ and $H_{A|BC}$) as outlined in Section 3. These entropies define the information distance and area using Equations (11) and (14), respectively. Then the QR for this qutrit system is defined to be the ratio of the weighted average of the perimeter of the triangle given by Equation (11)

(67) $${\langle P\rangle }_{{}_{M}}={\langle D_{AB}+D_{AC}+D_{BC}\rangle }_{{}_{M}}$$

to the weighted average of the area of the triangle given by Equation (14)

(68) $${\langle A\rangle }_{\;{}_{M}}:=\frac{1}{V_{\;{}_{M}}}\left(\int_{M}A\right),$$

where

(69) $$R:=\frac{{\langle P\rangle }_{{}_{M}}}{{\langle A\rangle }_{\;{}_{M}}}.$$

Here the averages are taken over all detector settings M with $V_{\;{}_{M}}={\left(2\pi^{2}\right)}^{2}$ for the area and $V_{\;{}_{M}}=\left(2\pi^{2}\right)$ for the perimeter. Without loss of generalization, we can fix *A*’s detector at $\theta_{a}=\varphi_{a}=0$ for all measurements. The integration that defines the reactivity will then be a function of the four Stokes parameters for *B* and *C*, namely $\theta_{b}$, $\varphi_{b}$ and $\theta_{c}$, $\varphi_{c}$, respectively.

**Figure 3:**
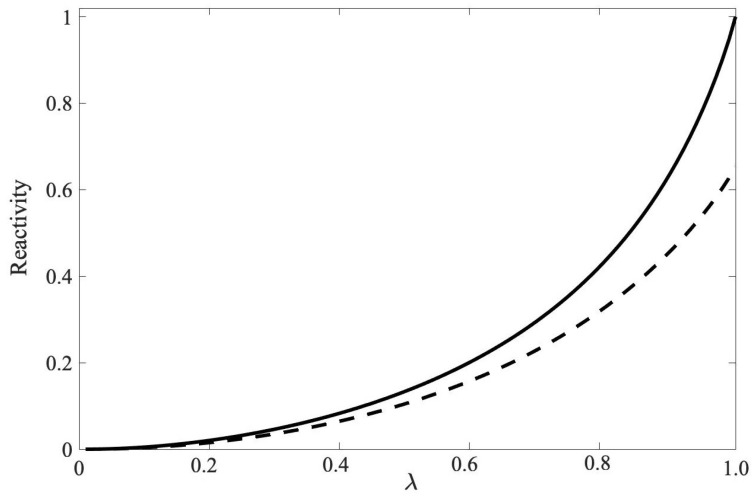
The QR is plotted as a function of the entanglement parameter λ for the three-qubit Werner state (**solid line**) defined by Equation (62), and the less correlated modified Werner state (**dashed line**) defined in Equation (64). The modified Werner state is formed using the |W〉 state instead of the maximally entangled qutrit |GHZ〉 state.

**Figure 4:**
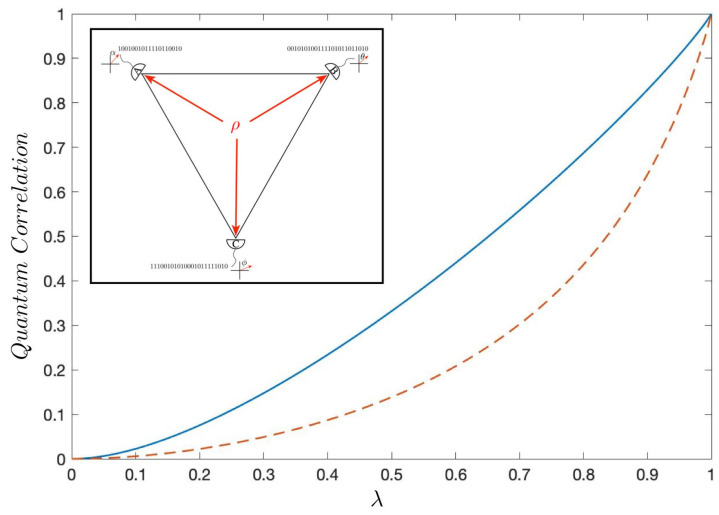
The QR (**dashed line**) and GQD (**solid line**) are plotted as a function of the entanglement parameter λ for the three-qubit Werner state defined by Equation (62).

Figure 3 illustrates the relationship of the QR for the Werner state and the modified Werner state. The curve for the modified Werner state in Figure 3 lies below the Werner state. This is intuitive as there is more entanglement in the |GHZ〉 state than in the |W〉 state. In Figure 4 we plotted the GQD and Reactivity for three-qubit Werner state.

### 4.3. QR for the Four-Qubit Werner State

QR is scalable in the number of qubits in the sense that it is an analytic function of the volume and its bounding surface. It is straight-forwardly extendable to higher dimensions. We demonstrate the scaling to a higher number of qubits by calculating the reactivity for a four-qubit state. In particular, we examine tin the section the four-qubit Werner state

(70) $$\rho_{Werner}=\lambda \;|GHZ_{4}\rangle \langle GHZ_{4}|+\frac{1-\lambda }{16}\;I,$$

where

(71) $$|GHZ_{4}\rangle =\frac{1}{\sqrt{2}}\left(|0000\rangle +|1111\rangle \right).$$

We will have four observers *A*, *B*, *C* and *E* measuring this state. They form a tetrahedron as illustrated in Figure 1. Once again, and without loss of generality, we can permanently align *A*’s detector along the *z*-axis and calculate the joint probability distributions $p_{ABCE}$ from the measurements on an ensemble of identically prepared states made by *B*, *C*, and *E*. From this distribution, we can define all the entropies as well as the information volume and the surface area for this tetrahedral geometry. This calculation mirrors the calculation we did in Section 4.2 except it is in one higher dimension. The QR for this four-qubit system is defined to be the ratio of the weighted average of the tetrahedrons surface to the weighted average of the tetrahedral volume. The weighted average of the surface is the sum of the weighted average of the four bounding triangle areas

(72) $${\langle S\rangle }_{{}_{M}}={\langle A_{ABC}+A_{ABE}+A_{ACE}+A_{BCE}\rangle }_{{}_{M}}.$$

where each of the weighted triangle areas is given bu Equation (14). The weighted average of the tetrahedral volume is given by Equation (15) and gives

(73) $${\langle V\rangle }_{\;{}_{M}}:=\frac{1}{V_{\;{}_{M}}}\left(\int_{M}V\right),$$

where $V_{\;{}_{M}}={\left(2\pi^{2}\right)}^{3}$. We obtain the reactivity for this qutrit state,

(74) $$R:=\frac{{\langle S\rangle }_{{}_{M}}}{{\langle V\rangle }_{\;{}_{M}}},$$

by using Equation (50) with d=4. Here the averages are taken over all detector settings relative to *A* that forms the “space of measurements” M. The integration that defines the reactivity will then be a function of the six Stokes parameters for *B*, *C* and *E*, namely $\theta_{b}$ and $\varphi_{b}$, $\theta_{c}$ and $\varphi_{c}$, and $\theta_{e}$ and $\varphi_{e}$, respectively.

**Figure 5:**
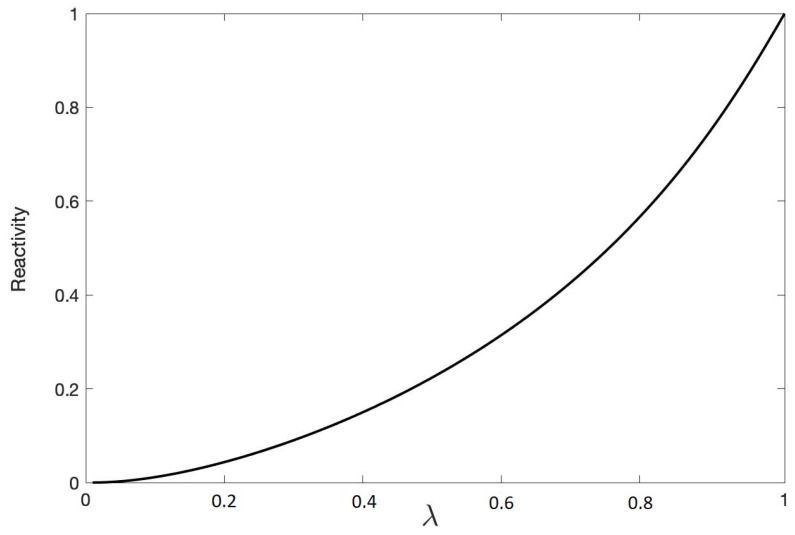
A plot of the reactivity (Equation (74) for the four-qubit Werner state (Equation (70) as a function of the entanglement parameter λ. The reactivity was scaled with an affine transform so that R(λ=0)=0 and R(λ=1)=1 This example illustrates the scalability of the reactivity and that it yields a monotonically increasing function in quantum correlation.

We showed that an increase in the entanglement and correlation parameter λ for the four-qubit Werner state yields correctly an increase in its degree of correlation as a function of λ. The reactivity in Equation (74) monotonically increases. This is illustrated in Figure 5. This is consistent with our results for the two-qubit example in Section 4.1 where an increase in entanglement parameter gives a corresponding increase in curvature.

## 5. Comparing Reactivity with Discord and Concurrence

**Figure 6:**
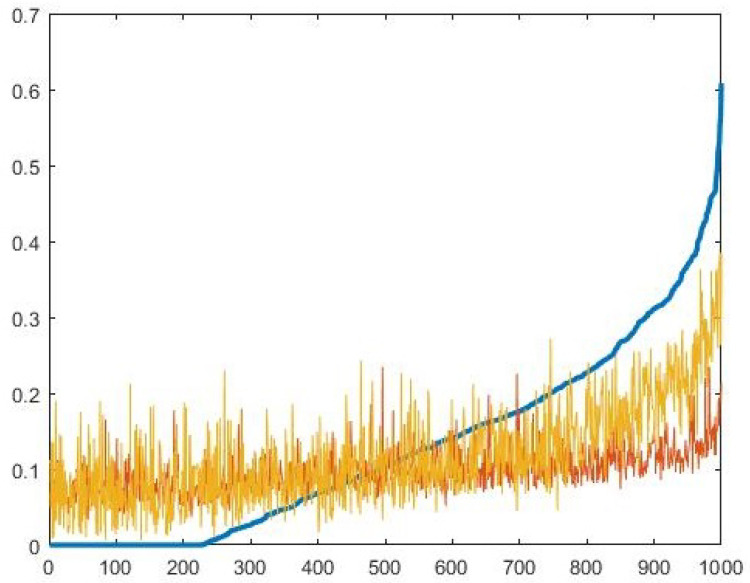
In this figure, the value of concurrence is shown by the blue line; QD is yellow; and QR is orange. The density matrices are sorted based on the values of concurrence in ascending order.

We looked at the values of QD, QR and concurrence for a random sample of density matrices consisting of 1000 random density matrices uniformly distributed according to Hilbert–Schmidt distance [31], then calculated the values of QD, QR, and concurrence for each density matrix, and sorted these density matrices in ascending order with respect to the value of concurrence. The result is illustrated in Figure 6.

As we see in Figure 6, both QD and QR have an increasing trend for increase in entanglement; nevertheless, states with zero concurrence might have higher or equal values of QD or QR compared to states with non-zero concurrence. Consequently QR and QD are not reliable measures of entanglement. This is not an unexpected result for QD as one can generate QD by local means of operation; on the other hand, one might expect QR, which is invariant under local unitary operators, to be a monotonic function of entanglement, but as we see in the Figure 6 this is not the case. Nevertheless, as we discussed in the previous chapters the purpose of QR and QD is not be a measure of entanglement but rather to give a notion of the presence of quantum correlation in systems with a higher number of qubits where calculating concurrence is not possible. It is also worth mentioning that QR can be used in applications which utilize QD, and QR can be used as a lower bound for QD. This is useful when we are dealing with large quantum systems where calculating QD is computationally challenging; in particular, QR is easier to calculate than QD and concurrence since it is based on Shannon’s entropy and does not require optimization.

## 6. Conclusions

We show in this paper that there is a relationship between quantum correlation and QR similar to the relationship using GQD. This geometric definition is based on measurement outcomes and gives us the opportunity to find a degree of quantum correlation emerging from its associated information geometry.

We show for the two-qubit Werner state that the QR qualitatively agrees with QD, although they are fundamentally different. In Figure 2 both QD and QR are measures for quantum correlation; however, it may increase under unitary LOCC in some cases. QR is invariant under unitary LOCC [22].

QR is defined by a weighted average of the surface-to-volume ratio over the space of measurements, and it appears to allow us to calculate a suitable degree of quantum correlation for higher-dimensional qudit states. It is scalable to multipartite systems in the sense that it can be extended to a larger number of qubits. It has the advantage of being interpretation free, unlike QD for multipartite states. It is a relatively straightforward analytic function of the joint probability distribution. QR does not require any global minimization procedure or matrix inversion. In other words, it appears to us to be relatively easy to calculate in comparison to other measures of correlation. However, the computational complexity of QR is dominated by the computation of the joint entropy over all the observers’ measurement outcomes and scales exponentially in *d* for a *d*-qubit quantum network. Nevertheless, this measure of quantum correlation appears to satisfy the properties required of such a measure, and perhaps it can be used as a mathematical tool to prove theorems in quantum computation.

There are many questions remaining for us to explore, e.g.,

- Is it possible to calculate QR for high fidelity by using a random configuration of detectors for large multipartite systems?
- Can we couple our choice of measurements conditioned on previous measurements to probe quantum entanglement in quantum networks?
- Can we examine coarse-grained measurements of our qudit network and its possible partitions?
- Can we use a neural network or artificial intelligence algorithm to probe optimal measurements to conduct on our quantum network?
- Is there a generalization of the quantum Sanov’s theorem that will give exponential convergence in the fidelity of our measure of correlation? [12,20]
- Do QD and QR capture the same aspects of quantum correlation?

Answering such questions would be important for quantifying the computational complexity of this method.
